# Development of a New Bead Movement-Based Computational Framework Shows that Bacterial Amyloid Curli Reduces Bead Mobility in Biofilms

**DOI:** 10.1128/JB.00253-20

**Published:** 2020-08-25

**Authors:** K. Malhotra, T. Hunter, B. Henry, Y. Ishmail, P. Gaddameedi, S. Tursi, Ç. Tükel, M. Hoffer, B. A. Buttaro, G. Queisser

**Affiliations:** a Department of Mathematics, Temple University, Philadelphia, Pennsylvania, USA; b Department of Microbiology and Immunology, Lewis Katz School of Medicine at Temple University, Philadelphia, Pennsylvania, USA; c Goethe Center for Scientific Computing, University of Frankfurt, Frankfurt, Germany; Université de Montréal

**Keywords:** *Escherichia coli*, *Enterococcus faecalis*, *Salmonella* Typhimurium, biofilm, curli

## Abstract

Biofilms exist in complex environments, including the intestinal tract, as a part of the gastrointestinal microbiota. The interaction of planktonic bacteria with biofilms can be influenced by material properties of the biofilm. During previous confocal studies, we observed that amyloid curli-containing Salmonella enterica serotype Typhimurium and Escherichia coli biofilms appeared rigid. In these studies, Enterococcus faecalis, which lacks curli-like protein, showed more fluid movement. To better characterize the material properties of the biofilms, a four-dimensional (4D) model was designed to track the movement of 1-μm glyoxylate beads in 10- to 20-μm-thick biofilms over approximately 20 min using laser-scanning confocal microscopy. Software was developed to analyze the bead trajectories, the amount of time they could be followed (trajectory life span), the velocity of movement, the surface area covered (bounding boxes), and cellular density around each bead. Bead movement was found to be predominantly Brownian motion. Curli-containing biofilms had very little bead movement throughout the low- and high-density regions of the biofilm compared to E. faecalis and isogenic curli mutants. Curli-containing biofilms tended to have more stable bead interactions (longer trajectory life spans) than biofilms lacking curli. In biofilms lacking curli, neither the velocity of bead movement nor the bounding box volume was strictly dependent on cell density, suggesting that other material properties of the biofilms were influencing the movement of the beads and flexibility of the material. Taken together, these studies present a 4D method to analyze bead movement over time in a 3D biofilm and suggest curli confers rigidity to the extracellular matrix of biofilms.

**IMPORTANCE** Mathematical models are necessary to understand how the material composition of biofilms can influence their physical properties. Here, we developed a 4D computational toolchain for the analysis of bead trajectories, which laid the groundwork for establishing critical parameters for mathematical models of particle movement in biofilms. Using this open-source trajectory analyzer, we determined that the presence of bacterial amyloid curli changes the material properties of a biofilm, making the biofilm matrix rigid. This software is a powerful tool to analyze treatment- and environment-induced changes in biofilm structure and cell movement in biofilms. The open-source analyzer is fully adaptable and extendable in a modular fashion using VRL-Studio to further enhance and extend its functions.

## Abstract (precis)

Mathematical models are necessary to understand how the material composition of biofilms can influence their physical properties. Here, we developed a 4D computational toolchain for the analysis of bead trajectories, which laid the groundwork for establishing critical parameters for mathematical models of particle movement in biofilms. Using this open-source trajectory analyzer, we determined that the presence of bacterial amyloid curli changes the material properties of a biofilm, making the biofilm matrix rigid. This software is a powerful tool to analyze treatment- and environment-induced changes in biofilm structure and cell movement in biofilms. The open-source analyzer is fully adaptable and extendable in a modular fashion using VRL-Studio to further enhance and extend its functions.

## INTRODUCTION

Bacterial biofilms are an integral part of human microbiota communities. The biofilms are exposed to multiple molecules such as nutrients and, in the case of pathogenic biofilms, antibiotics and monoclonal antibodies. In addition to chemicals, biofilms can interact with planktonic bacteria from the environment. These interactions can lead to recruitment into the multispecies biofilm (e.g., coaggregation in dental biofilms [1, 2], invasion leading to strain turnover in the community [e.g., bacteriocin-producing strains] [3, 4], or repulsion by active invasion resistance mechanisms [e.g., RbmA in *Vibrio*] [5]). Interactions with plasmid-containing planktonic cells also can result in the introduction of mobile genetic elements into the biofilm (6–9). The structure of the biofilm influences interactions with planktonic bacteria (2, 5).

The composition of the biofilm influences physical properties of the biofilms such as density and rigidity (10–13). Amyloid curli is characterized by its conserved, fibrillar cross-beta sheet structure and is expressed by enteric bacteria including Escherichia coli and Salmonella enterica serotype Typhimurium. Curli has multiple effects on biofilms (14, 15). Curli directly interacts with extracellular DNA (eDNA) and forms cellulose/curli structures (16, 17). Curli affects surface stiffness of the cells (18) and modifies the viscoelastic properties of the biofilm (19). Curli influences adherence to surfaces by providing high tensile strength binding to fibronectin and mediating prolonged firm attachment to glass surfaces (20). Curli also limits phage invasion into biofilms by binding to incoming phages (21).

In previous studies, using multitest well slides to analyze approximately 20-μm Enterococcus faecalis, E. coli, and *S*. Typhimurium biofilms by confocal microscopy, we consistently observed differences between the E. coli and *S*. Typhimurium (16, 22) compared to E. faecalis biofilms (this study). The *Enterobacteriaceae* species biofilms were very rigid, and low-density areas were easy to image. However, E. faecalis biofilms were hard to fix into place, and we had to make sure to apply pressure to the slide to have enough surface tension to prevent the cells from moving during imaging. Amyloid production has not been reported in enterococcal biofilms, although the pheromone cOB1 can form amyloid-like structures (23), nor did we observe any amyloid staining under our biofilm conditions using the same techniques we used to detect amyloids in *S*. Typhimurium (data not shown) (16).

Therefore, it was hypothesized that curli may be responsible for making the E. coli and *S*. Typhimurium biofilms more rigid under our biofilm culture conditions, reducing movement during confocal microscopy experiments. To test this hypothesis, a four-dimensional (4D) assay was designed to characterize physical properties of biofilms and how invading particles, such as bacteria, may interact with the biofilms with different physical properties. Small fluorescent beads have been used to probe macroscale-level material properties of biofilms (24–29). To examine bead movement on a microscale, 1-μm fluorescently labeled glyoxylate beads were added to biofilms, and movement was followed through a 10- to 20-μm biofilm over 10 to 20 min. A toolbox was developed to compute 4D trajectory length, bead velocity, trajectory-bounding box volume (minimal box containing the trajectory), bounding box density, and trajectory path visualizations.

## RESULTS

### Curli-containing biofilms have less movement than E. faecalis biofilms.

The physical properties of E. faecalis, E. coli, and *S*. Typhimurium biofilms, as well as curli isogenic mutants, were compared using 1-μm fluorescently labeled glyoxylate beads. These beads were chosen because of our long-term interest in modeling 4D bacterial movement through biofilms. However, beads were used for initial tool development to limit variability in bacterial cell size/arrangement (our main model organism, E. faecalis, also forms short chains) and possible confounding factors of E. faecalis surface components interacting with different species of bacterial cells. The beads are stable, have the same approximate size as E. faecalis, and provide a negatively charged surface mimicking bacterial cells (30, 31). Biofilms were prepared on no. 1.5 thick optical glass coverslips in 24-well plates. The biofilms were washed to remove autofluorescent growth medium and planktonic cells. One-micrometer fluorescently labeled glyoxylate beads (2 × 10^7^ beads in 1 ml phosphate-buffered saline [PBS]) were added to the biofilm and allowed to associate with the biofilm for 1 min. Unassociated beads were removed by washing, resulting in 40 to 140 beads associating with the biofilm, depending on its structure and composition. The coverslip inverted onto a coated slide with an approximately 25-μm coated well. To allow completely free movement above the biofilms, E. faecalis biofilms were also grown in 96-well optical-bottom plates. This was not technically feasible for the *Enterobacteriaceae* biofilms. Under the standard conditions used (13, 16), these biofilms form only at the air-liquid interface, and working distance for the confocal microscope objective made it difficult to image these biofilms formed in optical-bottom plates (please see Materials and Methods for details).

**FIG 1:**
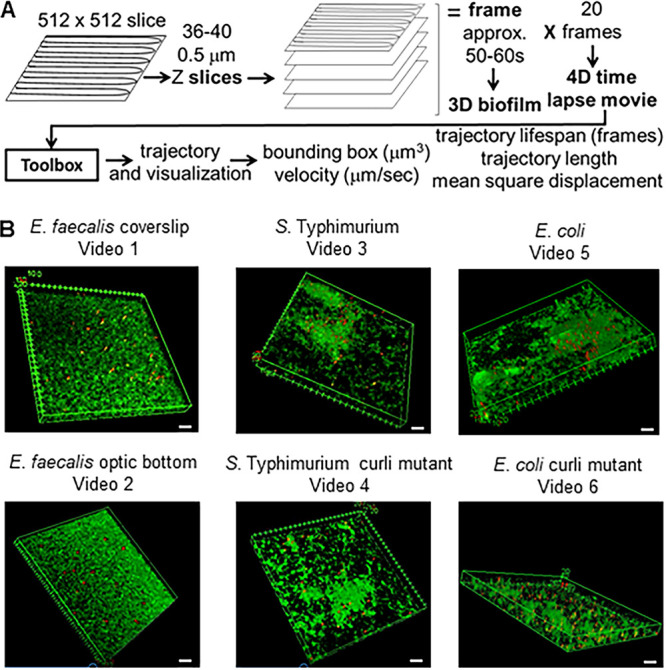
Overview of imaging method. (A) Biofilms are imaged on a Leica SP5 microscope using lower 512- by 512-pixel resolution. *z* slices are done in 0.5-μm steps. Most of the biofilms were between 18 and 20 μm thick, generating 36 to 40 *z* slices. The slices together made up a frame, which could be visualized as a 3D biofilm. Each 3D biofilm frame took 50 to 60 s to capture. This process was repeated 20 times to generate the 4D time-lapse video, which plays at approximately 500×. The data from the confocal.lif file were entered into the toolbox and trajectories were generated. The trajectories were visualized. Using the trajectories; bounding boxes (cubic micrometers), velocity (micrometers per second), association time (number of frames), and trajectory length (micrometers) were calculated. All biofilms, with the exception of E. faecalis optic bottom, were grown on coverslips. E. faecalis was grown in an optical-bottom 96-well plate. Areas containing thicker and thinner areas of the biofilm were chosen for imaging. Each image is one of the 20 3D images in the 4D assay. The scale bars are 20 μm. The biofilms analyzed are representative of at least three independent experiments.

Confocal microscopy was used to image 0.5 μm *z*-dimension slices through 18- to 20-μm sections of biofilm (Fig. 1). To image quickly enough to track bead movements, lower-resolution images (512 by 512 pixels; 0.48-μm pixel size) were taken, reducing the visual clarity of the time-lapse movies and images. Each 3D stack took approximately 1 min to capture. The image was generated 20 times (“frames”) for a total tracking time of 18 to 20 min (4D assay). Regions were chosen that contained a mixture of high- and lower-density biofilms. Three-dimensional time-lapse movies were generated for each biofilm using ImageJ (see Videos S1 to S6 in the supplemental material) (E. faecalis). Images of one time point of each of the 4D biofilm assays are shown in Fig. 1B. Total imaging times were approximately 18 to 20 min depending on biofilm thickness. The biofilm cells were visualized by SYTO 9 staining. SYTO 9 stains only the DNA of the cell usually present as a nucleoid in the center of the cell, so the visualization did not show the rest of the cell surrounding the DNA nor the matrix material, which may be present in the nonfluorescent areas of the biofilm. Sometimes beads would appear elliptically shaped. Rapid movement during the capture of the 40 *z*-position optical slices composing each 3D time point (frame) would cause the beads to be captured in sequential slices, giving the beads an elliptical shape in the compiled 3D image.

For curli-containing E. coli and *S*. Typhimurium biofilms, most beads remained associated with the biofilm, and the beads did not appear to have much movement (Videos S3 and S5, respectively). Beads in E. faecalis biofilms appeared to have greater overall movement. Movement of both the biofilm and the beads could be observed. Some of the beads appeared to make large movements and then slow down again, suggesting that the beads were disassociating and reassociating with the biofilm (Videos S1 and S2). To determine if sealing the coverslip onto the approximately 23- to 25-μm-deep multiwell slide (Video S3) was significantly affecting bead movement, E. faecalis biofilms were grown on optical-bottom 96-well plates (Video S2). The isogenic *S*. Typhimurium and E. coli curli mutant biofilms showed increased movement of the beads (Videos S5 and S6, respectively), but the beads appeared to move to a lesser extent than the E. faecalis beads.

### Development of bead movement analysis.

**FIG 2:**
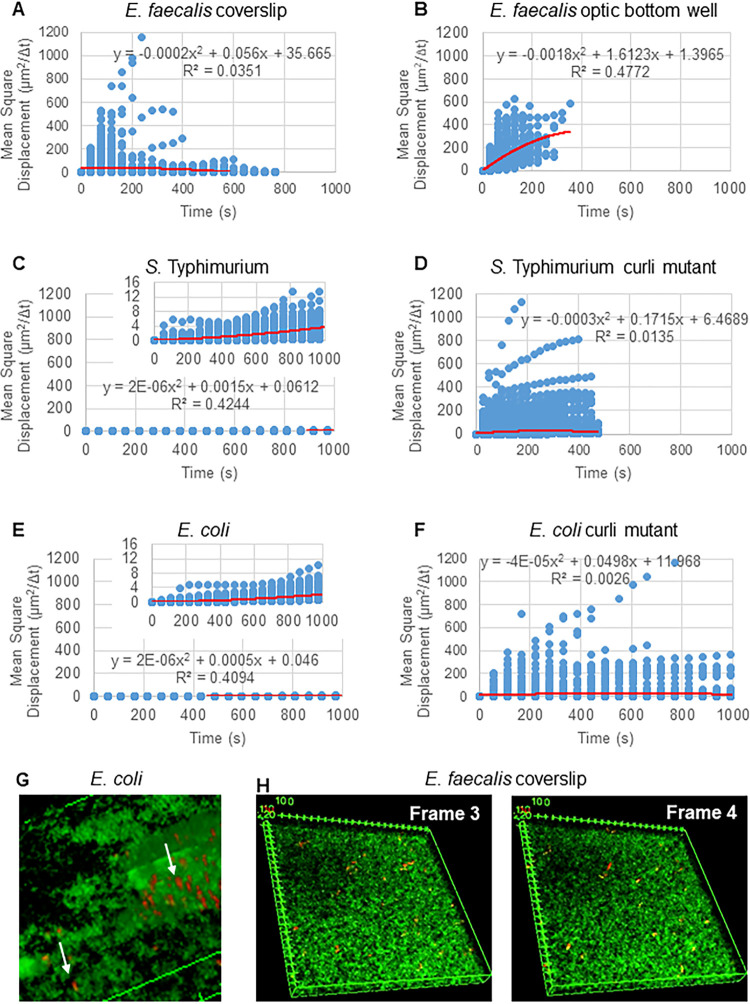
The bead movement in the biofilm is predominantly diffusive (Brownian motion). The following biofilms were grown on coverslips: E. faecalis (A), *S*. Typhimurium (C), E. coli (E), and isogenic curli mutants (D and F), and E. faecalis was grown in an optical-bottom 96-well plate (B). The evolution of the MSD over the course of each trajectory was calculated, and a quadratic least-squares fitting function used to determine if the average movement pattern of the beads was linear (red line), which would indicate movement by diffusive Brownian motion. (G) Example of elliptical bead movement observed in E. coli and *S. Typhimurium* biofilms taken from one frame of the E. coli 4D biofilm assay. (H) Example of large changes in bead patterns between frames taken from E. faecalis optical-bottom-well biofilm frame 3 and frame 4. Note that the biofilm itself also has some flowing movement (Video S2), giving the optical illusion of these frames not being in the same orientation.

The 3D confocal imaging data contain two channels, the red channel for glyoxylate beads and green channel for SYTO 9-labeled bacteria. To analyze bead movement in biofilms, the channels were first split into two tiff stacks using ImageJ (32). The ImageJ plug-in Mosaic (32) was used to compute the bead location in each 3D stack for all frames. The settings for Mosaic were chosen as follows. Particle detection used a radius of 3 pixels, a cutoff of 0.003, and Per/Abs of 0.12, and particle linking had a link range of 2, displacement of 10, and dynamics set to Brownian. Brownian motion was chosen because E. faecalis is a nonmotile bacterium, E. coli and *Salmonella* do not express flagella in biofilms, and the experiments are being done in a closed system in the absence of flow, suggesting that movement will unlikely be active directed movement. The choice of Brownian motion was confirmed by the computed mean square displacements (MSD) of the beads. Using the definition $\text{MSD}(m)=\frac{1}{m}\sum_{i=1}^{m}{\left(x_{i}-x_{0}\right)}^{2}$, where *m* is the number of trajectory segments, we compute the evolution of the MSD over the course of each trajectory. In a first approximation, if the graph of the MSD for a given trajectory is linear, then the movement of the bead can be assumed to be diffusive. Figure 2A to F show the MSD for all trajectories in the given biofilm. A quadratic least-squares fitting function was computed and used to demonstrate the average movement pattern of all the beads in the biofilm. As is apparent from these fitting functions, the trend is primarily linear in nature (with small nonlinear contributions), thus indicating diffusive behavior (Fig. 2A to F).

The Mosaic trajectory data were then stored in standard .csv files for further analysis. The trajectories computed by Mosaic are not necessarily unique to individual beads. If the confocal signal is too weak in one frame for Mosaic to identify the bead through image processing or if bead velocity is too high for Mosaic to predict the trajectory, the trajectory belonging to one bead may be registered as two or more shorter trajectories, especially in more fluid biofilms. The elliptically shaped bead movements (Fig. 2G) may be capable of producing short trajectories in multiple slices of the 40-slice 3D image. In *Enterobacteriaceae* biofilms, although the *x* and *y* movement is limited, rapid diffusion in the *z* direction may account for an increased number of small movement trajectories (800 for approximately 50 beads). Stitching trajectories between the 3D biofilms was not always feasible because bead movements were too large to be certain it was the same bead (see Fig. 2H for an example); thus, an unbiased manual stitching of the trajectories overall was not feasible. The computed trajectory lengths are therefore defined as the trajectory life span (number of frames the trajectory was followed). For all these reasons, the total number of trajectories exceeds the number of beads present in the biofilm and are an underestimation of the total bead association time.

In order to analyze the recorded trajectory data, a software tool was developed to compute trajectory lengths, trajectory life spans, trajectory-bounding box dimensions and volumes, average bead velocities and variances, weighted average velocities and variances, and averages/weighted averages and variances of bounding box volumes. In order to compute all weighted variables, the second imaging channel for SYTO 9-labeled bacteria was used to compute the local (within given trajectory-bounding boxes) cellular densities. The reason SYTO 9 DNA labeling was chosen, as opposed to using a cell membrane stain, was to give a more consistent signal to determine cellular density. Membrane staining intensity will vary depending on whether optic slice includes the top/bottom of a bacterial cell (signal across the entire cell) or cuts across a cell giving signal only around the outline of the bacterial cell. Determining the average cellular density around individual beads is motivated by the different length scales defined by cell size, trajectory lengths, and biofilm samples. This study compares the differences in cellular density, cellular density heterogeneity of different biofilms, and the effect on bead movement.

To guarantee reproducibility and tool usability, all methods were implemented in Groovy (http://groovy-lang.org) and Java (https://docs.oracle.com/en/java) within VRL-Studio (33), which allows modular workflow design with automatic user interface generation of all computational components (Fig. S1). The workflow design allows users to load Mosaic-generated trajectory .csv files and the two separate bead and bacteria tiff stacks. For each biofilm, the user is able to set *x*-, *y*-, and *z*-voxel (a three-dimensional pixel) dimension and the time interval between two recorded frames. The output is then written to .csv files. The bead trajectory software is published as an open-source tool on github (https://neurobox3d.github.io/Biofilm/).

### Curli reduces bead movement and may prolong bead interactions.

**FIG 3:**
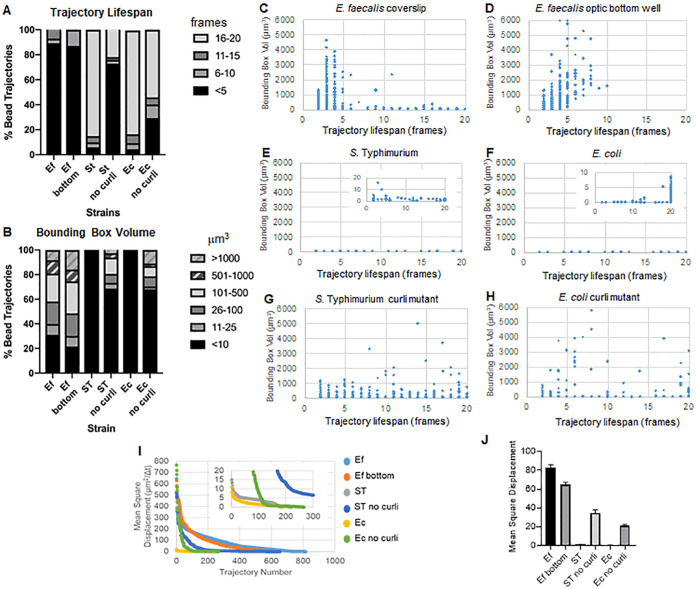
The presence of curli prolongs association time and reduces movement in biofilms. The following biofilms were grown on coverslips: E. faecalis (C), *S*. Typhimurium (E), E. coli (F), and isogenic curli mutants (G and H), and E. faecalis was grown in an optical-bottom 96-well plate (D). Association times are represented as trajectory times in numbers of frames and are presented as percent total bead trajectories (A) and scatter graphs (C to H). Bounding box volumes are presented as percent bead trajectories (A) and scatter graphs (C to H). (I) Comparison of the MSD of the beads in the different types of biofilms. (J) The average MSD of each type of biofilm. The bars indicate the 95% confidence interval of the data. Ef, *E*. faecalis; ST, *S*. Typhimurium; Ec, *E*. coli.

The bead trajectories were used to analyze bead movement. Movement in the biofilms over time was plotted as surface area covered (bounding box volume [cubic micrometers]), and trajectory life span with the biofilm was measured as the number of frames in which an individual trajectory was present (Fig. 3). The plots reflected what was seen visually. E. faecalis biofilms show more movement with bounding box values of 1 to 6,000 μm^3^ (Fig. 3A, C, and D). The movement is similar in biofilms grown on a glass coverslip and then inverted onto a coated slide with an approximately 25-μm coated well (Fig. 3C) versus biofilms grown in the bottom of optical glass wells and directly imaged (Fig. 3D). The only differences in the optical-bottom wells were that in E. faecalis biofilms, there seemed to be beads that appeared to be trapped near the glass coverslip interface, which registered as stable trajectories with life span times of >10 min with small bounding boxes, and a few beads had larger movements, suggesting that mounting the coverslip may have introduced some surface tension slowing the movement of some beads associated with very fluid areas of the biofilms (Fig. 3B to D and I). These trajectories were a very small percentage of the total trajectories, and, in fact, the average MSD of the E. faecalis was slightly lower than the mounted coverslip (Fig. 3). Beads were present visually in the E. faecalis biofilms during the entire time of imaging (Videos S1 and S2); however, no trajectory had a life span longer than 10 min, and many were between 2 and 5 min. This suggests that beads may move along a trajectory for a few frames (5 to 10 min), disassociate from the biofilm, and then reassociate with the biofilm. This would result in continuous presence of beads but only short life spans for each trajectory. Therefore, trajectory life spans are an underestimation of total association time of the beads with the biofilm.

Both of the *Enterobacteriaceae* biofilms (E. coli and *S*. Typhimurium) had smaller bounding box volumes of 0 to 10 μm^3^ (Fig. 3A, B, E, and F), and approximately 80% of the beads had trajectory life spans of 16 to 20 frames, which would be approximately 15 to 20 min (Fig. 3A, B, G, and H). Isogenic curli mutants showed increased movement with bounding boxes of 1 to 6,000 μm^3^ for E. coli and 1 to 5,000 μm^3^ for *S*. Typhimurium (Fig. 3A, B, F, and H). In contrast to E. faecalis biofilms, where more than 70% of the trajectories had bounding boxes of >10 μm^3^, only 30% of the *Enterobacteriaceae* species trajectories had bounding boxes of >10 μm^3^. The trajectory life span of curli mutants was reduced, but there were trajectories with significant movement that had longer trajectory life spans (Fig. 3H).

These results are consistent with the larger mean square displacements observed for the E. faecalis and curli mutant biofilms (Fig. 3I and J).

### The presence of curli shortens bead trajectory lengths and lowers velocities.

**FIG 4:**
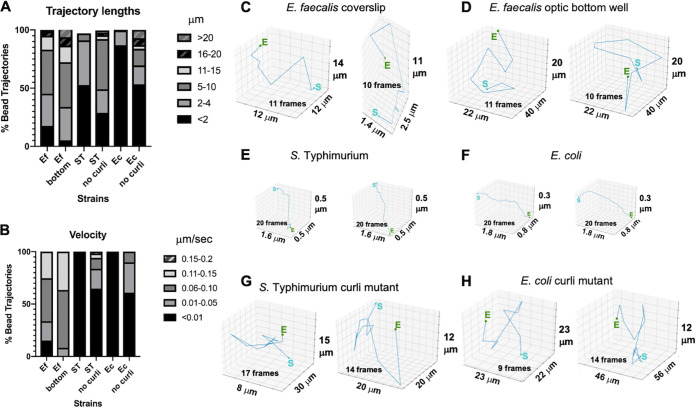
The presence of curli shortens trajectory lengths and trajectory paths can be visualized for beads moving in biofilms lacking curli. The following biofilms were grown on coverslips: E. faecalis, *S*. Typhimurium, E. coli, and isogenic curli mutants, and E. faecalis was grown in an optical-bottom 96-well plate. (A) Trajectory lengths are given in micrometers and are presented as percentages of total bead trajectories. (B) Velocities are shown in micrometers per second and are presented as percentages of total bead trajectories. Two randomly chosen long trajectories with movement are visualized for each strain for E. faecalis (C), *S*. Typhimurium (E), E. coli (F), and isogenic curli mutants (G and H), or in an optical-bottom 96-well plate for E. faecalis (B). The sizes of the bounding boxes on the *x*, *y*, and *z* axes are shown. The boxes are not drawn to scale. The number of frames is noted in the bounding box. For beads with movement in the E. faecalis biofilms and *Enterobacteriaceae* curli mutant biofilms, the longest trajectories with movement were often less than 20 frames.

Consistent with the smaller bounding boxes, beads in curli-containing biofilms had 10-fold shorter trajectory lengths of less than 4 μm versus trajectories of 5 to 20 μm that can be observed in biofilms lacking curli (Fig. 4A). The beads in curli-containing biofilms had up to 15-fold lower velocities, with most velocities less than 0.006 μm/s versus 0.01 to 0.15 μm/s in the absence of curli (Fig. 4B). Overall, curli mutant biofilms still had lower velocity and shorter trajectories than E. faecalis biofilms (Fig. 4A and B). Individual bead trajectories could be visualized. A few randomly chosen longer trajectories show various patterns and directions of bead movement (Fig. 4C to H). The lengths of the trajectories were shorter in biofilms lacking curli (Fig. 4A). Beads with longer trajectories did not have directly linear paths, which would be consistent with higher differences in velocity (approximately 15-fold greater) but slightly lower differences in trajectory length (approximately 10-fold greater). It is formally possible that movement is restricted in *x* and *y* planes but there is rapid movement up and down (*z* plane). The elliptical *z*-plane shapes (Fig. 2G) and high number of trajectories (800) coming from approximately 50 beads suggest that this may be the case.

### Bead movement is not entirely dependent on cellular density.

**FIG 5:**
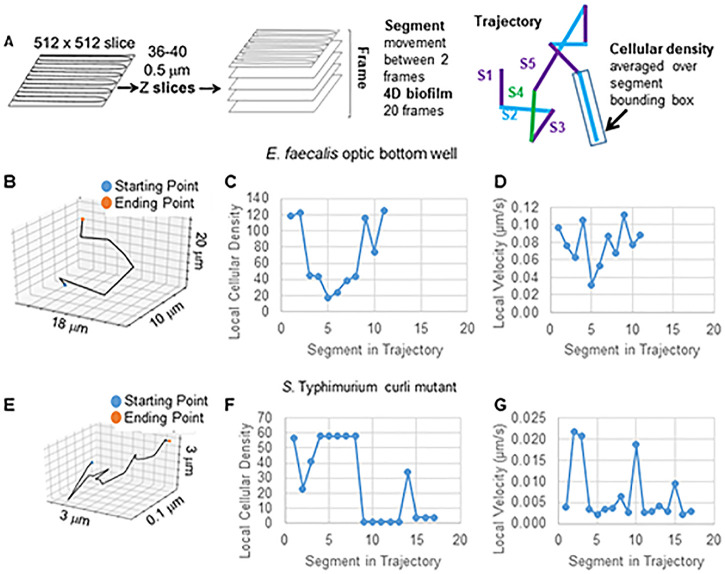
Trajectory segment analysis to correlate velocity and cellular density. (A) Diagram of trajectory segment analysis. (B) Example of randomly chosen long trajectory from E. faecalis optical-bottom-well biofilm. (C and D) Segment-by-segment plot of local cellular density (C) and velocity (D). (E) Example of randomly chosen long trajectory from *S*. Typhimurium biofilm. (F and G) Segment-by-segment plot of local cellular density (F) and velocity (G).

The bead trajectories were tracked across 20 3D images. The shortest trajectory life span was 2, corresponding to a bead followed between only two 3D biofilms. The longest trajectory life span was 20, corresponding to a bead followed through 20 3D biofilms and composed of 19 trajectory segments (Fig. 5A). To determine if bead movement was dependent on cellular density, the cellular density was calculated using the green fluorescent protein (GFP) intensity per voxel. The density around each segment of the bead trajectory could be determined as a locally averaged density. Two examples of the segment-by-segment analysis of the local density and local velocity of two longer-trajectory life span beads, one from E. faecalis on optical-bottom plates and one from the *Salmonella* curli mutant, are shown in Fig. 5B, D, and E to G.

**FIG 6:**
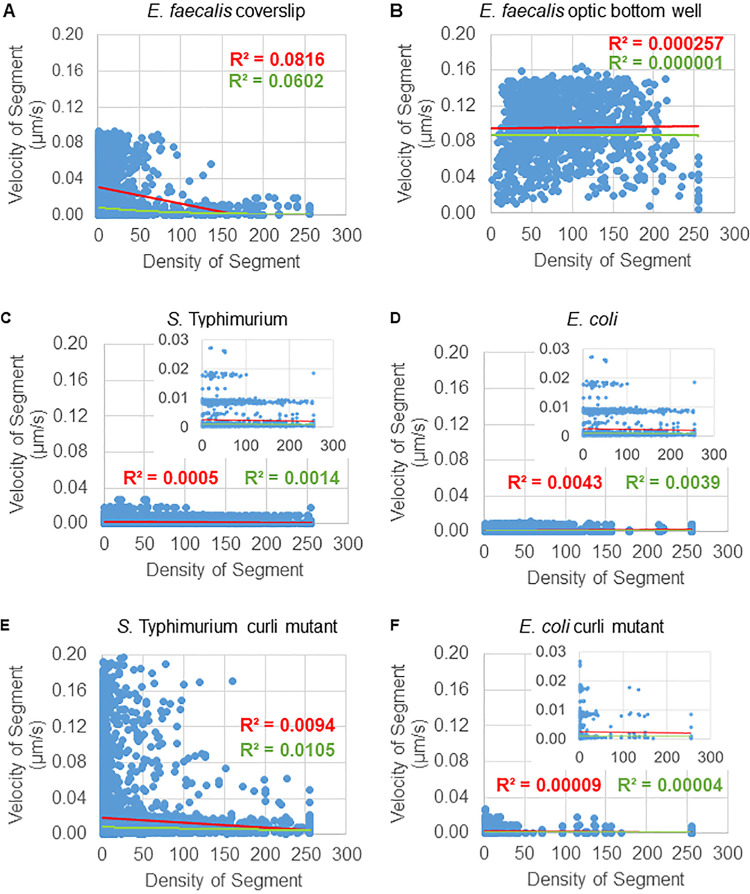
Bead velocity is not entirely dependent on biofilm density. The following biofilms were grown on coverslips: E. faecalis (A), *S*. Typhimurium (C), E. coli (D), and isogenic curli mutants (E and F), and E. faecalis was grown in an optical-bottom 96-well plate (B). Trajectories were analyzed on a segment scale (Fig. 5). For each segment, bead velocity was plotted against bounding box cellular density (average GFP per voxel within the bounding box). The red line is the linear regression, and the green line is the exponential regression.

Density dependence was evaluated on two scales. On the segment scale, the average density around each trajectory segment was calculated, and the local segment bead velocity was weighted by the segment density (Fig. 5A). On the trajectory scale, the previously computed locally weighted velocities from the segment scale were averaged over the entire trajectory. On both scales, some density dependence for certain biofilms could be observed. The segment scale results are shown in Fig. 6, and the corresponding trajectory scale data are found in the supplemental data. E. faecalis biofilms, when grown on a glass coverslip and inverted onto a multiwell slide (Fig. S2), showed a visible density dependence (Fig. 6A), while the E. faecalis, grown on the bottom of a 96-well plate (Fig. 6B), did not. These data suggest that the highly fluid E. faecalis biofilm may be compacted slightly by mounting on a multiwell slide and are consistent with slight loss of faster-moving beads (Fig. 3C versus Fig. 3D). Furthermore, *Salmonella* and E. coli biofilms (Fig. 6C and D) showed minor to no density dependence. Finally, the curli mutant biofilms (Fig. 6E and F) showed little to no density dependence. Averaging the density over the bounding box of the full trajectory was too coarse to resolve this effect (Fig. S2).

## DISCUSSION

A significant difference in the behavior enterococcal biofilms was observed compared to previous studies on E. coli and *Salmonella* biofilms (16, 22). The enterococcal biofilms appeared to be more flexible and have more movement. It was hypothesized that these differences may influence how particles interact with these biofilms. To test this hypothesis, we used a 4D assay using 3D time-lapse confocal microscopy to examine the behavior of 1-μm glyoxylate beads in biofilms. Software was developed to analyze bead trajectories, which were computed with the Mosaic plug-in for ImageJ. Movement of the particles was tracked to determine bead trajectories, trajectory life span (as determined by the number of frames the bead trajectory was followed), bounding box dimensions to measure total area through which the bead traveled, and velocity of bead movement and to visualize individual bead trajectories. Based on these data, it was concluded that curli reduces bead movement and velocity as well as influences length of association for a subpopulation of beads. Bead movement in the biofilm is not strictly density dependent and is influenced by material properties of the biofilm.

The conclusion that curli reduces bead movement is supported by the larger bounding boxes and higher velocity of the bead movement in E. faecalis biofilms and isogenic curli *csgBA* mutants (curli protein and nucleator protein mutants) (34, 35) (Fig. 3 and 4). For *S*. Typhimurium and E. coli biofilms, in very low-density regions of the biofilm, when the bead was clearly not sitting on the bottom of the slide but was suspended in the matrix material, the beads did not move. Curli has been shown to be present in low-density regions of E. coli and *Salmonella* biofilms, including regions lacking cells (16). Curli amyloids are rigid beta sheet molecules (35). They bind to DNA and change its structure (16, 36). Therefore, it is reasonable to predict that curli may be influencing the material properties of the biofilm by binding to the eDNA and making it more rigid. Biofilms produced by *Salmonella* curli mutants were more robust than biofilms produced by E. coli curli mutants. Further studies are required to determine the structural components accounting for these observed differences.

Beads with longer times of trajectory life spans can be seen in both high- and low-density areas of cells in curli-containing biofilms with beads (Fig. 3). In biofilm movies of E. faecalis biofilms and the curli-mutant enteric biofilms, beads visually stay associated with the biofilm the entire time the biofilm was being imaged (see Videos S1 and S2 in the supplemental material). However, the ImageJ plug-in Mosaic does not have the ability to stitch trajectories. This has two effects. First, very rapid bead movement cannot be tracked as a single trajectory by the software. This results in many more trajectories than beads actually present in the biofilm. For use of this software for tracking long trajectories, e.g., cells moving into a biofilm to transfer a plasmid, the shorter trajectories can be trimmed from the data. However, ways to stitch the elliptically shaped bead movements seen in the *Enterobacteriaceae* biofilms will be important to quantitate this type of movement. Further development of stitching capabilities will be useful because beads can be seen in the E. faecalis biofilms for all 20 min, yet 90% of the trajectories have a time of association of less than 5 frames (Fig. 3B). Therefore, the beads overall may stay associated with the biofilm, but they may briefly disassociate and move to a new location beyond our current capabilities to track as the same bead. In this newer, slightly more distant location, the bead may establish a new short-interval track. This kind of shift may not be easily visually detected, although careful visual observation shows inconsistent tracks of the beads associated with the biofilm for 20 min. Regardless, in curli-containing biofilms, many beads clearly stay in the same location throughout the time course of imaging (Videos S3 and S4; Fig. 3B).

Movement of the beads in E. faecalis biofilms as well as E. coli and *S*. Typhimurium did not appear to significantly correlate with bacterial cellular density. In our assay, biofilm bacterial density is based on the number of bacteria present as visualized by SYTO 9 staining. Density calculations were based on the average relative intensity of the GFP signal in the bounding box, and the visualization did not show the remaining parts of the cell surrounding the DNA. The influence of the density on bead movement was analyzed by comparing the density within each bounding box to the velocity with which the bead traveled and the distance the bead traveled (trajectory length). If the bead movement was density dependent, in lower-density regions beads would travel at greater velocity and for greater distances, and in higher-density regions the opposite would be true. Plots comparing these values, though, did not show these trends universally (Fig. 5), indicating that bead movement is not heavily influenced by cellular density and that some other phenomena are contributing to bead movement. The density method in these studies did not take into account uneven distribution of the cells in the bounding box (e.g., bead on the edge of a clump of cells) and any differences in the density of the matrix materials. Furthermore, continued refinements in the image analysis will be combined with matrix staining during future studies on how material properties of the biofilm influence movement.

The cause of bead movement within the biofilm is not fully determined. There is no flow in the system. The trajectory paths of randomly chosen beads have different patterns (Fig. 4C to I, Fig. 5B, and Fig. 3E), suggesting that there is no strong flow pulling beads in a similar direction. Additionally, the computed mean square displacements indicate passive diffusion, which can be influenced by various properties of the biofilm. Overall, bead movement in E. faecalis biofilms was similar when trajectories were observed on coverslips inverted on approximately 25-μm wells and sealed with fingernail polish compared to biofilms growing on optical-bottom wells that are placed directly on the objective (Fig. 3 and 4). Heating by the lasers is unlikely to generate the movement given the observed physical trajectory lengths compared to the length scale of the heat equation. Movement is also observed in the biofilm in general. Biofilms have been demonstrated to behave on the bulk scale like viscoelastic fluids, as might be expected from an amalgam of weakly interacting entangled polymers and cells (37–39). These bulk scale material properties are determined by the nature of those biofilm components and their interactions. These material properties could include differences in rigidity (e.g., curli bound to eDNA) as well as differences in electrostatic and hydrophobic interactions between the negatively charged beads and the biofilm cells and matrix materials. Interestingly, the fibrillar structure of curli (40) may limit movement side to side (*x* and *y* planes) but allow for rapid movement up and down in the *z* plane (Fig. 2G). Further study and tool development will be required to determine if this is the case. The charge of the beads may also contribute to the movement. The negatively charged 1-μm beads were chosen because they mimic the negatively charged zeta potential of bacterial cells such as E. faecalis (a major focus of our research) (30, 41). E. faecalis isolates from biliary stents have different zeta potentials, making this an interesting avenue of future investigation, as these tools are expanded to examine bacterial cell invasion into biofilms (41). The movement of the beads may be influenced by movement of the biofilm (moving at the same rate as the biofilm), independent movement based on electrostatic interaction of the beads with the matrix material, or a combination of both. The material properties in biofilms are likely spatially heterogeneous (42). The tools developed in these studies will allow future determination of how macroscale viscoelastic fluidity may influence microscale biofilm properties, including bead and cell movements within the biofilms.

We have developed a new 4D tool to analyze particle movement in 3D biofilms over time, including the trajectory-bounding box (minimal box containing the trajectory), trajectory path, velocity, and trajectory life span. This tool was used to generate data that, taken together, suggest that the presence of the amyloid curli in *Enterobacteriaceae* biofilms reduces bead movement in the biofilm and prolongs the interaction times of the beads independent of cell density. The tool has multiple possible applications for biofilm analysis. We originally used the bead technique for Tükel lab to determine changes in biofilm structure upon treatment with monoclonal anticurli antibodies (22). Future studies will be done in our laboratories to determine how movement of biofilms may influence interactions with plasmid-containing cells and their subsequent transfer of plasmids as well as implications for the movement of bacteria through complex microbiota communities with various material properties.

## MATERIALS AND METHODS

### Bacterial strains and growth conditions.

E. faecalis OG1RF was grown in Todd-Hewitt (TH) medium. Ongoing studies in the laboratory support use of TH as a medium for multispecies biofilms. *Enterobacteriaceae* strains were S. enterica serotype Typhimurium strain ATCC 14028 and its isogenic *csgBA* mutant as well as E. coli UTI89 and its isogenic *csgBA* mutant. Overnight cultures were grown in Luria broth, and the wild-type *S*. Typhimurium was grown in 50 μg/ml nalidixic acid.

### Biofilm growth.

Enterococcus faecalis biofilms were grown either in 24-well plates on flame-sterilized 12-mm no. 1.5 optical glass coverslips or in 96-well no. 1.5 optical-bottom glass plates (MatTek, Ashland, MA). Biofilms were grown for 24 h at 37°C. For *Enterobacteriaceae* biofilms, sterilized 12-mm no. 1.5 glass coverslips were placed in 24-well plates containing 700 μl of media. LB no-salt broth and LB low-salt broth were used to support maximal curli production of the *S*. Typhimurium and E. coli biofilms, respectively. Wells were inoculated with a 1:100 dilution of overnight cultures of wild-type *S*. Typhimurium, E. coli, or the isogenic *csgBA* mutants. For production of curli, biofilms were grown at 28°C for 6 to 8 days at a slant to allow for a biofilm to attach to the coverslip and form on the air-liquid interface. Optical-bottom microtiter plates were not used for *Enterobacteriaceae* because they grow as a pellicle at the air-liquid interface. Even if the plates are grown at a slant to allow an attachment point at the bottom when the plates are laid flat on the microscope stage, only the thick, adhered biofilm was attached to the optical-glass bottom and the pellicle biofilm, with thick and thin patches, still floated above the glass. The resulting focal planes were above the working depth of the confocal objective. Carefully placing the biofilm coverslip on a slide allowed the biofilm to be imaged on a single surface.

### Confocal imaging.

For imaging, the biofilms were washed 2 times with PBS to remove planktonic cells, and, in the case of TH, the medium has autofluorescence that is detected in the green channel. Crimson 1-μm glyoxylate FluoSpheres beads (Molecular Probes) were diluted 1:50 in PBS, and 1 ml was added to the washed biofilm. Beads were incubated on the coverslip for 1 min. Beads were removed, the biofilm was washed 1 time with 1 ml PBS, and the glass coverslip was inverted onto a multiwell plate containing a 1-μl drop of SYTO 9 (green fluorescent DNA stain; diluted in accordance with the manufacturer’s instructions). The coverslip was sealed, with care being taken not to press down on the coverslip. E. faecalis biofilms grown on optical-bottom wells were treated the same except SYTO 9 was added with beads for 1 min incubation. The biofilm was washed 1 time with PBS to remove excess beads not associated with the biofilm, and fresh PBS was added and left in the well for the duration of the imaging.

Imaging was done on a Leica SP5 microscope equipped with a TCS confocal system using a 63× objective and 512- by 512-pixel resolution (Fig. 1). SYTO 9 was excited with a 488-nm laser, and emission was measured from 495 to 540 nm. Crimson (red) beads were excited with a 633-nm laser, and emission from 650 nm to 700 nm was measured.

### Image analysis.

Leica .lif files (Leica image file format) were imported into ImageJ, and 3D overlay movies were generated using the 3D viewer plug-in (43). Trajectory computation and analysis were done using the software toolchain developed for this publication (see “Development of bead movement analysis” in Results). The automated toolchain is available as open-source software on github (https://neurobox3d.github.io/Biofilm/). Data analysis was done using Microsoft Excel and Prism.

## Supplementary Material

Supplemental file 1

Supplemental file 2

Supplemental file 3

Supplemental file 4

Supplemental file 5

Supplemental file 6

Supplemental file 7
