# Supplementary material for: Development of a New Bead Movement-Based Computational Framework Shows that Bacterial Amyloid Curli Reduces Bead Mobility in Biofilms
Source: J Bacteriol. 2020 Aug 25;202(18):e00253-20. doi: 10.1128/JB.00253-20 (PMC7925071; doi:10.1128/JB.00253-20)
Supplement: Supplemental file 1 [file JB.00253-20-s0001.pdf]

Supplemental Material

Development of a new bead movement based computational framework shows curli amyloids reduce bead mobility in biofilms.

K. Malhotra[a], T. Hunter*[b], B. Henry[a*], Y. Ishmail[b], P. Gaddameedi[b], S. Tursi[b], Ç. Tükel[b], M. Hoffer[c], B. A. Buttaro[b*], and G. Queisser[a]

Supplement Video Figure Legends

Figure S1. 4D video of *E. faecalis* coverslip biofilm. *E. faecalis* OG1RF biofilms were grown on 1.5 thick optic glass coverslips for 24 hrs. Biofilms are imaged on a Leica SP5 Microscope using lower 512x512 resolution. Z slices were done in 0.5 μm steps. The slices together made up a frame, which could be visualized as a 3D biofilm. Each 3D biofilm frame took 50-60s to capture. This process was repeated 20 times to generate the 4D time lapse video, which plays at approximately 500X. Video is representative of at least 6 independent experiments.

Figure S2. 4D video of *E. faecalis* optic bottom plate biofilm. *E. faecalis* OG1RF biofilms were grown 96-well optic bottom plates for 24 hrs. Biofilms are imaged on a Leica SP5 Microscope using lower 512x512 resolution. Z slices were done in 0.5 μm steps. The slices together made up a frame, which could be visualized as a 3D biofilm. Each 3D biofilm frame took 50-60s to capture. This process was repeated 20 times to generate the 4D time lapse video, which plays at approximately 500X. Video is representative of 3 independent experiments.

Figure S3. 4D video of *S.* Typhimurium coverslip biofilm. *Salmonella enterica* serotype Typhimurium biofilms ATCC 14028 biofilms were grown on 1.5 thick optic glass coverslips for 6-7 days. Biofilms are imaged on a Leica SP5 Microscope using lower 512x512 resolution. Z slices were done in 0.5 μm steps. The slices together made up a frame, which could be visualized as a 3D biofilm. Each 3D biofilm frame took 50-60s to capture. This process was repeated 20 times to generate the 4D time lapse video, which plays at approximately 500X. Video is representative of 3 independent experiments.

Figure S4. 4D video of *S.* Typhimurium curli mutant coverslip biofilm. *Salmonella enterica* serotype Typhimurium biofilms ATCC 14028 curli (*csgBA*) mutant were grown on 1.5 thick optic glass coverslips for 6-7 days. Biofilms are imaged on a Leica SP5 Microscope using lower 512x512 resolution. Z slices were done in 0.5 μm steps. The slices together made up a frame, which could be visualized as a 3D biofilm. Each 3D biofilm frame took 50-60s to capture. This process was repeated 20 times to generate the 4D time lapse video, which plays at approximately 500X. Video is representative of 3 independent experiments.

Figure S5. 4D video of *E. coli* coverslip biofilm. *E. coli* UTI89 were grown on 1.5 thick optic glass coverslips for 6-7 days. Biofilms are imaged on a Leica SP5 Microscope using lower 512x512 resolution. Z slices were done in 0.5 μm steps. The slices together made up a frame, which could be visualized as a 3D biofilm. Each 3D biofilm frame took 50-60s to capture. This process was repeated 20 times to generate the 4D time lapse video, which plays at approximately 500X. Video is representative of 3 independent experiments.

Figure S6. 4D video of *E. coli* curli mutant coverslip biofilm. *E. coli* UTI89 curli (*csgBA*) were grown on 1.5 thick optic glass coverslips for 6-7 days. Biofilms are imaged on a Leica SP5 Microscope using lower 512x512 resolution. Z slices were done in 0.5 µm steps. The slices together made up a frame, which could be visualized as a 3D biofilm. Each 3D biofilm frame took 50-60s to capture. This process was repeated 20 times to generate the 4D time lapse video, which plays at approximately 500X. Video is representative of 3 independent experiments.

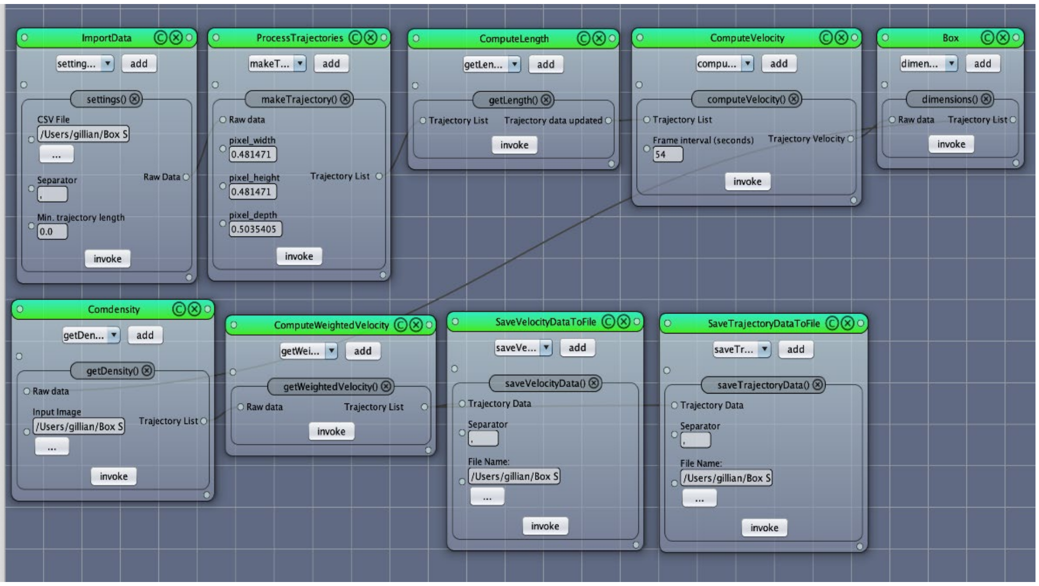

Figure S7. Bead trajectory evaluation with VRL-studio toolbox. The imaging file (in these studies a Leica .lif file) and the x, y, and z-voxel dimensions as well as the time interval is entered into the toolbox. The trajectories, trajectory lengths, trajectory velocities, bounding box, bounding box density, weighted velocity (velocity adjusted for density) are computed and placed in a .csv file that can be imported into data analysis programs such as Microsoft Excel.

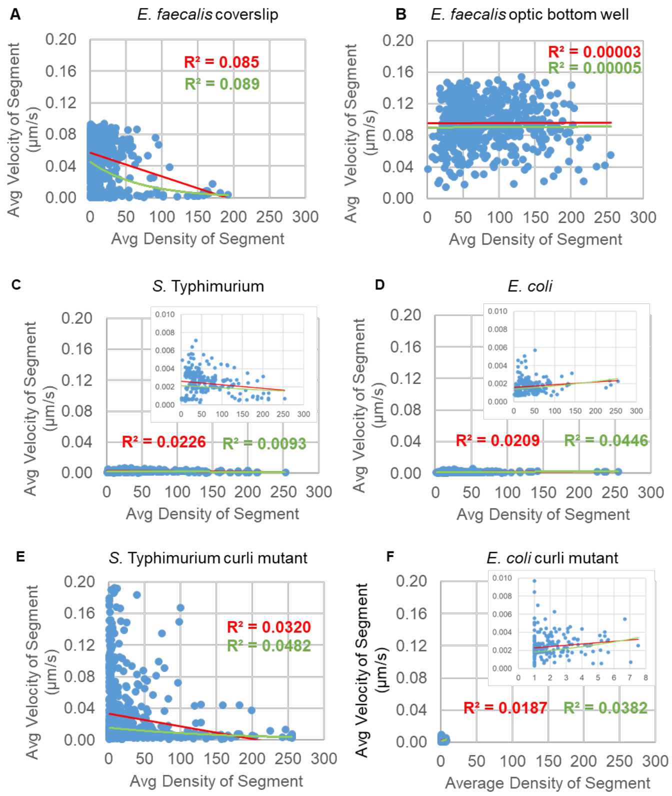

Figure S8. Bead velocity is not dependent on biofilm density. Biofilms were grown on coverslips *S.* Typhimurium (5A), *E. coli* (5B) and *E. faecalis* (5C) isogenic curli mutants (5E and 5F) or in an optic bottom 96-well plate (*E. faecalis* bottom, 5D). Trajectories were analyzed on a trajectory scale. Bead velocity in µm/sec was plotted against bounding box cellular density (average GFP per voxel within the bounding box). The red line is the linear regression and the green line is the exponential regression.
